# AAZTA^5^/AAZTA^5^-TOC: synthesis and radiochemical evaluation with ^68^Ga, ^44^Sc and ^177^Lu

**DOI:** 10.1186/s41181-019-0068-1

**Published:** 2019-08-01

**Authors:** Jean-Philippe Sinnes, Johannes Nagel, Frank Rösch

**Affiliations:** Johannes Gutenberg-University Mainz, Institute of Nuclear Chemistry, Fritz-Strassmann-Weg 2, 55128 Mainz, Germany

**Keywords:** Gallium-68, Scandium-44, Lutetium-177, AAZTA^5^-TOC, AAZTA^5^, TOC, Somatostatin, PET, Theranostic

## Abstract

**Purpose:**

AAZTA (1,4-bis (carboxymethyl)-6-[bis (carboxymethyl)]amino-6-methylperhydro-1,4-diazepine) based chelators were initially developed in the context of magnetic resonance imaging. First radiochemical studies showed the capability of AAZTA to form stable complexes with radiolanthanides and moderately stable complexes with ^68^Ga. For a systematic comparison of the labelling capabilities with current diagnostic and therapeutic trivalent radiometals, AAZTA^5^ (1,4-bis (carboxymethyl)-6-[bis (carboxymethyl)]amino-6-[pentanoic-acid]perhydro-1,4-diazepine) was synthesized representing a bifunctional version with a pentanoic acid at the carbon-6 atom. To evaluate the effect of adding a targeting vector (TV) to the bifunctional chelator on the complex formation, AAZTA^5^-TOC was synthesized, radiolabeled and tested in comparison to the uncoupled AAZTA^5^.

**Methods:**

AAZTA^5^ was synthesized in a 5-step synthesis. It was coupled to the cyclic peptide TOC (Phe^1^-Tyr^3^ octreotide) via amide bound formation. AAZTA and AAZTA^5^-TOC complex formations with ^68^Ga, ^44^Sc and ^177^Lu were investigated at different pH, temperature and precursor amounts. Stability studies against human serum, PBS buffer, EDTA and DTPA were performed.

**Results:**

AAZTA^5^ and AAZTA^5^-TOC achieved quantitative labelling (> 95%) at room temperature in less than 5 min with all three nuclides at pH ranges from 4 to 5.5 with low precursor amounts of 1 to 10 nmol. [^44^Sc]Sc-AAZTA^5^ complexes as well as [^44^Sc]Sc-AAZTA^5^-TOC were completely stable. The ^177^Lu complexes of AAZTA^5^ and AAZTA^5^-TOC showed high stability comparable to the ^44^Sc complexes. In contrast, the [^68^Ga]Ga-AAZTA^5^ complex stability was rather low, but interestingly, [^68^Ga]Ga-AAZTA^5^-TOC was completely stable.

**Conclusion:**

AAZTA^5^ appears to be a promising bifunctional chelator for ^68^Ga, ^44^Sc and ^177^Lu with outstanding labelling capabilities at room temperature. Complex stabilities are high in the case of ^44^Sc and ^177^Lu. While [^68^Ga]Ga-AAZTA complexes alone lacking stability, [^68^Ga]Ga-AAZTA^5^-TOC demonstrated high stability. The latter indicates an interesting feature of [^68^Ga]Ga-AAZTA^5^–labelled radiopharmaceuticals.

## Introduction

The ongoing development in molecular imaging is focusing more and more on theranostic approaches as they link imaging directly with therapy as well as monitoring of the treatment (Baum and Kulkarni 2012; Baum and Rösch 2013; Rösch et al. 2017). In this context, patient-individual dosimetry is important for application of long-lived therapeutic nuclides. This emphasizes the advantage of a longer-lived PET-nuclide like ^44^Sc over ^68^Ga (Khawar et al. 2018; Roesch 2012a; Kerdjoudj et al. 2016).

Most of the commonly used nuclides for PET imaging and therapy are trivalent metals Me(III), thus have a positive charge of 3 as ions in solution. The most frequently used bifunctional chelators (BFC) are macrocyclic derivatives based on DOTA and NOTA. Bifunctionalization of DOTA normally uses one of the acid groups to connect to the targeting vector (TV). DOTA-conjugated TV’s are typically ^68^Ga-labeled at ca. 95 °C for 10–15 min to achieve high labelling yields (Tsionou et al. 2017; Price and Orvig 2014; Roesch 2012b). NOTA- or NODAGA-conjugated analogues allow ^68^Ga-labelling at lower temperature, if larger amounts of the labelling precursor are utilized, yet for many compounds at lower concentration heating to 95 °C is common to achieve fast and quantitative labelling (Eisenwiener et al. 2002). For theranostic strategies with ^177^Lu only DOTA can be used, since NOTA derivatives are not suitable for complexing ^177^Lu. Again, ^177^Lu-labellig of DOTA-peptides is achieved at temperatures close to 100 °C and optimal labelling pH is around 4.

In addition, some relevant molecular targeting vectors (e.g. based on proteins) are temperature and pH sensitive. Accordingly, optimal chelators for radiolabeling sensitive biomolecules with trivalent radiometals (^68^Ga, ^44^Sc, ^177^Lu, ^90^Y, ^213^Bi, ^225^Ac) at room temperature and pH 5–6 would be extremely important. There are some proper candidates for labelling at room temperature. HBED (Schuhmacher et al. 1992), THP (Berry et al. 2011), H_2_dedpa (Boros et al. 2010, 2012), DATA (Waldron et al. 2013; Seemann et al. 2017; Farkas et al. 2017; Seemann et al. 2015) show the desired labelling capabilities mostly for ^68^Ga, while THP and DATA have optimum labelling parameters reaching fast quantitative yields at room temperature on wide pH ranges up to pH 6 (Tsionou et al. 2017). AAZTA^5^/AAZTA^CN^ were reported to provide good labelling with not only ^68^Ga, but also ^44^Sc and ^177^Lu (Pfister et al. 2015; Nagy et al. 2017; Manzoni et al. 2012).

In this work, we want to evaluate the AAZTA lead structure in several aspects. First, a bifunctional derivative (AAZTA^5^), needed to later covalently attach the chelator to a TV, was synthesized. Here, our strategy is to replicate the approach we successfully introduced for the DATA chelator (Seemann et al. 2017). Second, we systematically optimize radiolabeling protocols for Me(III)-AAZTA^5^ complexes with ^68^Ga, ^44^Sc and ^177^Lu. Third, we correlate labelling yield with Me(III)-AAZTA^5^ complex stability in vitro. Fourth, we synthesized a proof-of-principle radiopharmaceutical, namely AAZTA^5^-TOC, to again determine radiolabeling efficiency and in vitro stability.

## Materials and methods

### Synthesis of AAZTA^5^

**Fig. 1:**
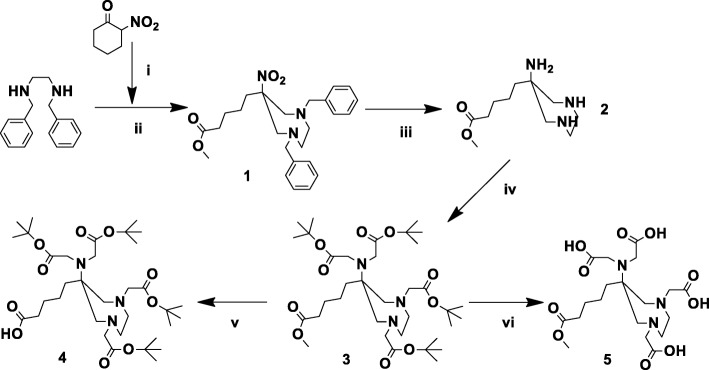
Synthesis of the bifunctional chelator AAZTA^5^: (i) A21, MeOH, in situ; (ii) paraformaldehyde, MeOH, yield 85%; (iii) Pd (OH)_2_/C, EtOH, H_2_, not isolated; (iv) *tert*-butyl-bromoacetate, K_2_CO_3_, ACN, yield (iii/iv) 55%; (v) LiOH, dioxane/water, yield 80%; (vi) 90% TFA/5% H_2_O/5% triisopropylsilane (TiS), yield 60% after HPLC

All standard chemicals were acquired from Sigma-Aldrich, Merck and VWR. AAZTA^5^ (2,2′-(6-(bis (carboxymethyl)amino)-6-(4-carboxybutyl)-1,4-diazepane-1,4-diyl) diacetic acid) was synthesized in a 5-step synthesis. The reactions to form the diazepine ring 1 via ring opening of nitrocyclohexanon and the full reduction to 2 has been published before as it is also part of the DATA synthesis (Fig. 1) (Seemann et al. 2017). To form the fully alkylated AAZTA, the ongoing reaction was catalyzed by KI and moderate temperatures of 40 °C as well as a huge excess of tert-butyl bromoacetate. By doing so reaction, yields could be pushed to 50–60%. Analytics of 3 lead to: ^1^H-NMR (CDCl_3_, 400 MHz, δ [ppm]): 3.65 (s, 4 H); 3.61 (s, 4 H); 3.22 (s, 3 H); 2.99 (d, J = 14.1 Hz, 2 H); 2.85–2.65 (m, 4 H); 2.63 (d, J = 14.1 Hz, 2 H); 2.31 (t, J = 7.4 Hz, 2 H); 1.62–1.52 (m, 4 H); 1.44 (s, 18 H); 1.43 (s, 18 H); 1.25 (m, 2 H); ^13^C-NMR (CDCl_3_, 100 MHz, δ [ppm]): 174.37 (s); 172.89 (s); 170.94 (s); 80.86 (s); 80.38 (s); 65.29 (s); 63.17 (s); 62.61 (s); 59.39 (s); 52.09 (s); 51.56 (s); 37.34 (s); 34.26 (s); 28.31 (s); 28.25 (s); 25.89 (s); 21.83 (s). To deprotect the bifunctional acid group, the methyl ester is cleaved with LiOH in dioxane/water to afford 4, while for the evaluation of the free chelator 5 the protective *tert*-butyl groups were cleaved with TFA leaving the methyl ester intact.

### Synthesis of AAZTA^5^-TOC

A standard amide coupling was performed to couple the AAZTA^5^ to TOC. The TOC (Phe^1^-Tyr^3^-octreotide) was purchased from ABX Germany as H-D-Phe-Cys-Tyr (^t^butyl)-D-Trp (boc)-Lys (boc)-Thr (^t^butyl)-Cys-Thr (ol) protected for amide coupling on the N-terminal amine of D-phenylalanine. As coupling reagent HBTU with an excess of HOBt was added to a solution of 4 in DMF. DIPEA was used as organic base. The formation of the active ester could be observed on LC-MS (column used: ZORBAX with 5% to 95% ACN (+ 0.1% MeCOOH)/water (+ 0.1% MeCOOH) in 5 min) as the mass of 4 (ESI-MS (M + H^+^): 672.45, 673.45, 674.46) with a retention time of 3.4 min changed to the active ester (ESI-MS (M + H^+^) with a retention time of 3.8 min. Monitoring the formation of the active ester showed full formation of the active ester after 15–20 min and the TOC was added. After coupling, all protective groups were cleaved with TFA. HPLC purification was performed on a LUNA column (Phenomenex® Luna® 10 μm C18(2) 100 Å) with isocratic conditions of 21% ACN (+ 0.1% TFA)/79% water (+ 0.1% TFA). The final product AAZTA^5^-TOC was collected at a retention time of 15–17 min in an yield of 45% over the coupling reaction, deprotection and purification. ESI-MS showed the pure product at 1464,6 (M + H)^+^ (C_67_H_94_N_13_O_20_S_2_ predicted 1464,62).

### Radiolabeling with ^68^Ga, ^44^Sc and ^177^Lu

For radiolabeling with ^68^Ga a ^68^Ge/^68^Ga generator (TiO_2_-based matrix, Cyclotron Co., Obninsk, Russia) was used with acetone post-processing separating iron and zinc impurities as well as ^68^Ge breakthrough (Zhernosekov et al. 2007). Radiolabeling with ^44^Sc was performed with a ^44^Sc/^44^Ti generator (Filosofov et al. 2010; Pruszyński et al. 2010) located at the Institute of Nuclear Chemistry in Mainz. ^177^Lu was provided by itg Munich following the carrier-free production pathway ^176^Yb(n,γ)^177^Yb → ^177^Lu (Lebedev et al. 2000).

Radiolabeling for all radiometals was performed in 1 ml of 0.2 M ammonium acetate buffer pH 4.5 and 5.5. Due to the post processing for ^44^Sc the nuclide is provided in 0.25 M ammonium acetate buffer pH 4 (Pruszyński et al. 2010) and for first studies the pH was not adjusted above pH 4. With 1 ml labeling volume for ^68^Ga and ^44^Sc precursor concentrations of 5 and 10 pmol/l and for ^177^Lu lower concentrations of 0.5 and 1 pmol/l were used. To show the mild labelling capability of the AAZTA^5^ chelator, the reaction temperature was adjusted to 25 °C with a BT 03 heater from HLC BioTech (Germany). Labelling studies were performed with 50 MBq for ^44^Sc and 100 MBq for ^68^Ga and ^177^Lu. At different time points of 1, 3, 5 and 10 min aliquots for TLC and HPLC analytics were taken. The pH was controlled at start of labelling and after labelling, was finished.

For reaction control TLC (TLC Silica gel 60 F_254_ Merck®) with citrate buffer (pH 7) and ammonium acetate buffer (pH 7)/MeOH 50/50 was used and compared to radio HPLC (Chromolith flush column, water: ACN with 0.1% TFA, 5 to 95% ACN in 10 min). TLCs were measured in RITA TLC imager (Elysia Raytest). The citrate TLC showed free radio metal with an Rf of 0.9 and all labelled compound sticked to an Rf of 0.1 to 0.3. As TLC would show colloidal radiometals sticking to an Rf of 0.1 to 0.2 as labelled compound, radio-HPLC was used to exclude the presence of colloidal radiometals, as colloidal radiometals cannot be eluted from the used HPLC columns.

### Stability studies

Stability studies were performed in HS, PBS and EDTA/DTPA solution (pH adjusted to 7 by PBS buffer) in triplicate, starting from Me(III)-AAZTA^5^/ AAZTA^5^-TOC batches of > 95% radiochemical purity. Time points were adjusted to the nuclides half-life: for ^68^Ga 0.5, 1, 2 h; for ^44^Sc 0.5, 1, 4, 8 h, 24 h; for ^177^Lu 0.5, 1 and 2 h, 1 and 7 days. Stabilities against HS were also tested for adsorption to HS as fractions of the stability study at given time points were added to acetonitrile. After precipitation and centrifugation, the supernatant solution was removed and residue was measured for activity. HS (human male AB plasma, USA origin) was bought from Sigma Aldrich, PBS was prepared with a BupH™ Phosphate Buffered Saline Pack (PIERCE), EDTA and DTPA solution were prepared using the prepared PBS buffer by adding EDTTA and DTPA to a 0.01 M concentration.

## Results

### Synthesis of AAZTA^5^ and AAZTA^5^-TOC

The established synthesis for DATA^5m^ and DATA^5m^-TOC (Seemann et al. 2017) was successfully transferred to AAZTA^5^ and AAZTA^5^-TOC. The key step, attaching four *tert*-butyl-acetate-arms to the diazepine backbone, was optimized to work in good yields by forcing the reaction with slightly increased temperatures of 40 °C and the addition of KI. Without the exchange from bromine to iodine on the *tert*-butyl bromoacetate nearly no alkylation was observed. Even with KI the reaction is still relatively slow and needs to be stirred over two days. Deprotection of the methyl group on the AAZTA^5^ was done similar to that of DATA^5m^. Purification by extraction (dichloromethane against 0.1 M NaCO_3_ in water) gave the pure product ready for coupling in the dichloromethane phase. Ready for coupling AAZTA^5^ could be isolated with a yield of 35% calculated over all reaction steps from the *N,N′*-dibenzylethylenediamine used in the first reaction step.

Formation of the AAZTA^5^ active ester with HBTU (in DMF with 7 eq of DIPEA) was fast and could be monitored by LC-MS showing the active ester. After 15 min and positive LC-MS control, the active ester was added to the TOC solved in DMF. After one night, the DMF was removed and residues were resolved in 95% TFA/2.5% water/2.5% TiS (triisopropylesilane) to start the deprotection step. Lower concentrations of TFA with dichloromethane as solvent were not acidic enough to fully deprotect the chelator, leaving 1–2 *tert-*butyl groups even after 1 night. After 3–4 h of reaction the TFA were removed and the residue solved in 80% water/20% acetonitrile for HPLC. Yields after the HPLC were in a solid range of 30–40%.

### Radiochemical evaluations with ^68^Ga

**Fig. 2:**
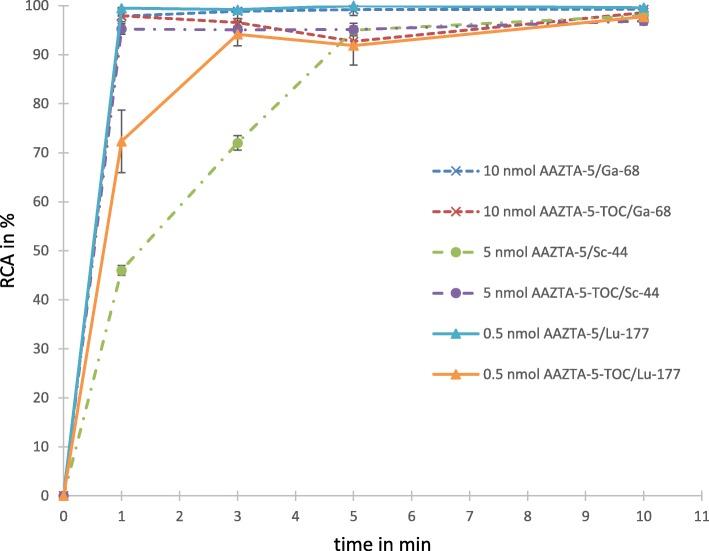
Labelling kinetics for lowest precursor amounts to reach reproducible quantitative yields within 10 min at room temperature

For both the free chelator AAZTA^5^ as well as the AAZTA^5^-TOC, radiolabelling with 10 nmol precursor showed an almost quantitative yield of > 98% in less than 5 min; most of the time reaching > 95% even after 1 min at room temperature (Fig. 2). At 5 nmol of precursor amount, a slower kinetic was observed, but still yielding > 90% after 15 min most of the time, yet reproducibility became a problem. Variation of the pH from 4.5 to 5.5 showed no difference in the kinetics or the overall yield. Thus, 10 nmol was found to be the optimal precursor amount to prepare [^68^Ga]Ga-AAZTA^5^ and [^68^Ga]Ga-AAZTA^5^-TOC, ready to be investigated in subsequent stability studies.

Stability of [^68^Ga]Ga-AAZTA^5^ against HS and PBS were high for 1 h, but decreased slowly at 2 h to a value of 85% and 79%, respectively. Adding EDTA or DTPA decreased the stability further up to 40–50% after 2 h. In contrast to the free chelator, the [^68^Ga]Ga-AAZTA^5^-TOC was completely stable against HS, PBS, EDTA and DTPA over two hours (Table 1).

**Table 1:** Radiolabelling data for [^68^Ga]Ga-AAZTA^5^, [^68^Ga]Ga-AAZTA^5^-TOC, [^44^Sc]Sc-AAZTA^5^, [^44^Sc]Sc-AAZTA^5^-TOC after 10 min (15 min for 5 nmol AAZTA^5^) at room temperature

	^68^Ga-Radiolabelling				^44^Sc-Radiolabelling	
	AAZTA^5^		AAZTA^5^-TOC		AAZTA^5^	AAZTA^5^-TOC
pH	4.5	5.5	4.5	5.5	4.0	4.0
5 nmol	92.3 ± 1.8	89.8 ± 2.4	94.3 ± 1.7	91.7 ± 3.5	98.0 ± 0.6	96.9 ± 0.9
10 nmol	98.5 ± 0.8	99.1 ± 0.5	99.0 ± 0.7	98.6 ± 0.9	99.5 ± 0.3	96.7 ± 1.2

### Radiochemical evaluation with ^44^Sc

Labelling both AAZTA^5^ and AAZTA^5^-TOC with ^44^Sc showed a quantitative yield of > 97% with 10 nmol in less than 5 min, most of the time reaching > 95% even after 1 min, all at room temperature. Lowering the precursor amount to five nmol induced slower kinetics for the free chelator, still yielding > 95% after 10 min for AAZTA^5^. In contrast, radiolabelling with five nmol of AAZTA^5^-TOC was always > 95% in less than 5 min at room temperature. Labelling with five nmol was stable enough to use it for stability studies. Stability studies showed for both the free chelator as well as the AAZTA^5^-TOC stability against HS, PBS, EDTA and DTPA over 8 h. [^44^Sc]Sc-AAZTA^5^-TOC remained stable with > 90% in HS and PBS even over 24 h (Table 2).

**Table 2:** Stability data for ^68^Ga, ^44^Sc and ^177^Lu complexes of AAZTA^5^ and AAZTA^5^-TOC against human serum, PBS buffer and EDTA and DTPA in PBS buffer in percent of stable complex at specific time points (% labelled compound compared to free activity found at given time point)

	^68^Ga			^44^Sc			^177^Lu		
	time	AAZTA^5^	AAZTA^5^-TOC	time	AAZTA^5^	AAZTA^5^-TOC	time	AAZTA^5^	AAZTA^5^-TOC
HS	30 min	93.0 ± 0.8	98.1 ± 0.2	1 h	93.7 ± 0.9	95.1 ± 0.6	1 h	99.8 ± 0.1	92.5 ± 1.9
	1 h	92.7 ± 1.3	96.9 ± 0.2	4 h	94.3 ± 1.7	94.3 ± 0.8	2 h	99.7 ± 0.1	92.3 ± 2.6
	2 h	85.3 ± 3.4	95.0 ± 0.3	8 h	93.2 ± 1.1	95.0 ± 1.1	24 h	99.9 ± 0.1	91.5 ± 2.1
				24 h	91.3 ± 1.8	93.8 ± 1.0	7 d	82.5 ± 3.2	86.3 ± 3.2
PBS	30 min	97.5 ± 1.0	99.1 ± 0.2	1 h	95.3 ± 0.9	97.3 ± 0.5	1 h	97.8 ± 0.5	92.0 ± 1.4
	1 h	88.7 ± 0.5	99.2 ± 0.1	4 h	92.7 ± 1.3	98.5 ± 0.9	2 h	98.6 ± 0.4	93.5 ± 1.3
	2 h	78.5 ± 4.5	99.3 ± 0.1	8 h	95.9 ± 1.2	96.7 ± 1.4	24 h	99.1 ± 0.6	91.0 ± 2.2
				24 h	96.0 ± 0.8	94.7 ± 1.6	7 d	96.0 ± 0.8	93.1 ± 1.8
EDTA	30 min	93.0 ± 0.2	98.9 ± 0.4	1 h	93.7 ± 1.3	96.0 ± 0.4	1 h	98.9 ± 0.2	95.7 ± 2.4
	1 h	50.3 ± 7.4	99.4 ± 0.2	4 h	90.0 ± 3.2	97.6 ± 0.7	2 h	97.9 ± 0.3	95.1 ± 2.6
	2 h	49.5 ± 2.5	99.6 ± 0.1	8 h	92.3 ± 2.1	96.9 ± 1.0	24 h	79.2 ± 2.9	86.8 ± 2.6
				24 h	91.9 ± 1.8	95.4 ± 2.1	7 d	48.9 ± 1.5	77.5 ± 2.3
DTPA	30 min	84.3 ± 0.9	97.8 ± 0.2	1 h	94.3 ± 0.9	98.7 ± 0.3	1 h	99.1 ± 0.2	98.9 ± 0.5
	1 h	78.0 ± 1.4	99.4 ± 0.1	4 h	92.5 ± 0.5	98.9 ± 0.7	2 h	99.1 ± 0.1	96.2 ± 3.2
	2 h	45.7 ± 1.3	99.6 ± 0.3	8 h	93.8 ± 1.2	96.9 ± 1.5	24 h	80.4 ± 5.4	82.8 ± 4.7
				24 h	93.4 ± 1.1	92.6 ± 3.2	7 d	59.0 ± 14.3	72.9 ± 5.6

### Radiochemical evaluation with ^177^Lu

General labelling procedures for DOTA conjugated radiopharmaceuticals with n.c.a. ^177^Lu showed that DOTA is capable of complexing ^177^Lu in molar ratios from 1: 5 to 1: 10 for save reproducible quantitative labelling. 100 MBq of ^177^Lu is around 0.1 nmol. ^177^Lu-labelling with 0.5 nmol AAZTA^5^ (ratio 1: 5) showed quantitative yields of > 98% already after 1 min at room temperature. Formation of [^177^Lu]Lu-AAZTA^5^-TOC had lower kinetics, yet also leading to yields of > 95% after 5 min. Both showed rapid labelling with ^177^Lu in less than 1 min at one nmol precursor. Labelling behaviour was the same for pH 4.5 and 5.5. Labelling of 500 MBq ^177^Lu with 5 nmol precursor (for stability studies) were also performed yielding > 98% after 1 min reaction time at room temperature (Table 3).

**Table 3:** Radiolabelling data for [^177^Lu]Lu-AAZTA^5^ and [^177^Lu]Lu-AAZTA^5^-TOC

	^177^Lu-Radiolabelling			
pH	4.5		5.5	
ligand: Lu ratio	AAZTA^5^	AAZTA^5^-TOC	AAZTA^5^	AAZTA^5^-TOC
5: 1	97.9 ± 1.6	98.9 ± 0.5	99.9 ± 0.2	97.7 ± 1.5
10: 1	98.6 ± 0.8	98.7 ± 1.1	99.3 ± 0.7	99.6 ± 0.1

Stability studies showed for both the free chelator as well as the AAZTA^5^-TOC good stabilities of > 90% against HS and PBS over 24 h. Stabilities against EDTA and DTPA were slightly lower with > 85% after 24 h. Longer studies of 7 d proved good stability against PBS with > 90% and a small degradation in HS with > 85% (mostly adsorption on serum proteins, visible by precipitation). 7 d stability values for EDTA and DTPA gave increasing instability down to 75 and 70% respectively for AAZTA^5^-TOC and to 60 and 50% respectively for the free chelator.

## Discussion

Our experience concerning the synthesis of DATA^5m^-TOC (Seemann et al. 2017) could be transferred, with adjustments on the alkylation step, to prepare the free chelator AAZTA^5^ and its TOC derivative AAZTA^5^-TOC in good yields. Radiolabelling with ^68^Ga showed good and rapid complexation at room temperature with quantitative yields in less than 5 min for both the free chelator as well as the AAZTA^5^-TOC. The complexation also worked on pH 5.5 without showing issues forming colloidal ^68^Ga, giving the same high yields after 5 min as for pH 4.5. This proves that elevation of the pH does not affect the labelling in a negative way. This rapid labelling at room temperature represents a significant advantage of AAZTA^5^ over DOTA. Second, fast labelling kinetics of DOTA also demand higher temperatures and precursor amounts and this demonstrates the most relevant feature of AAZTA^5^ compared to DOTA-derivatives.

Stability of the free [^68^Ga]Ga-AAZTA^5^ complex was not 100% over the 2 h, releasing 10–15% of the ^68^Ga against HS and PBS consistent with literature (Waldron et al. 2013; Baranyai et al. 2013). However, and extremely interesting, [^68^Ga]Ga-AAZTA^5^-TOC was completely stable over two hours against HS and PBS, showing a positive effect of the TOC on the stability of the ^68^Ga-complex. Due to the short spacer between the chelator and the TV, this could be a steric influence of the peptide moiety, but also the contribution of the amide formed in the coupling process may influence ^68^Ga coordination. Adding EDTA or DTPA to the PBS increased the instability for AAZTA^5^, whereas AAZTA^5^-TOC remained fully stable. It appears to be necessary to study the impact of moieties other then TOC to further understand this phenomenon.

Rapid and quantitative ^44^Sc-AAZTA^5^ complex formation (both AATZA^5^ and AAZTA^5^-TOC) at room temperature with low precursor amounts of five nmol demonstrate that the AAZTA^5^ structure is almost ideal for labelling ^44^Sc. With 100 MBq, ^68^Ga in contrast to 50 MBq ^44^Sc the effective amount of radiometal used per labelling was even higher for ^44^Sc due to its longer half-life. In fact, 50 MBq ^44^Sc contain near double the nmol of radio metal compared to 100 MBq ^68^Ga. Using half of the precursor amount on double the nmol radio metal leads to a chelator to radio metal ratio in the labelling solution that is 4 times lower in the ^44^Sc labelling than for ^68^Ga one. In addition, less precursor is also lowering the chelator concentration in the labelling solution. Consequently, AAZTA^5^ and AAZTA^5^-TOC have outstanding labelling capabilities for ^44^Sc. [^44^Sc]Sc-AAZTA^5^ and [^44^Sc]Sc-AAZTA^5^-TOC were completely stable against HS and PBS over two times the half-lives of ^44^Sc and even stable after 24 h. Only the addition of EDTA and DTPA showed a small release of the ^44^Sc after 24 h while the complexes are stable over 8 h. Combining the rapid quantitative radiolabelling with the good stability, AAZTA^5^ and AAZTA^5^-TOC offer optimal labelling capabilities for ^44^Sc.

Radiolabelling with ^177^Lu was performed in equimolar ratios showing that even a ratio of 1: 5 between radiometal and chelator gave quantitative yields. Using ratios of 1: 10, rapid labelling is observed after one minute already at room temperature. Adjusting the pH to 5.5 had no influence on labelling speed or yields showcasing the same stable labelling as seen before with ^68^Ga and ^44^Sc. Stability over 24 h showed both the AAZTA^5^ as well as the AAZTA^5^-TOC to be stable against HS and PBS with a slight decomplexation against ETDA and DTPA. After seven days, both complexes stay intact against PBS whereas the degradation by addition of EDTA and DTPA increases. For HS, some adsorption to serum proteins was observed by precipitation, while most of the complex stays intact. Overall, the complex stability is good for PBS and needs further evaluation in vivo to see the influence of the data from the HS.

## Conclusion

The chimerical triaaza-type AAZTA^5^ chelator showed rapid quantitative radiolabelling at room temperature with low precursor amounts up to pH 5.5 for ^68^Ga, ^44^Sc and ^177^Lu. Attaching TOC forming the AAZTA^5^-TOC had no influence on the labelling proving the good labelling capabilities can be transferred. The ^68^Ga complex of AAZTA^5^ was not stable as predicted, but AAZTA^5^-TOC showed complete stability revealing a strong positive effect of the TV on the stability. This effect needs further research. The [^44^Sc]Sc-AAZTA^5^ complex as well as [^44^Sc]Sc-AAZTA^5^-TOC were completely stable followed by the ^177^Lu complexes being stable over 24 h with small degradation after one week. AAZTA^5^ appears to be an excellent chelator for mild rapid labelling of both ^44^Sc and ^177^Lu forming a theranostic pair.

## Data Availability

Data sharing not applicable to this article as no datasets were generated or analyzed during the current study.

## Abbreviations

ACN: Acetonitrile
BFC: Bifunctional chelators
DMF: Dimethylformamide
e.g.: Exempli gratia
EtOH: Ethanol
HPLC: High performance liquid chromatography
HS: Human serum
MeOH: Methanol
PBS: Phosphate buffered saline
PET: Positron Emission Tomography
TFA: Trifluoroacetic acid
TiS: Triisopropylsilane
TLC: Thin-layer chromatography
TV: Targeting vector

## References

[CR1] Baranyai Z, Uggeri F, Maiocchi A, et al. Equilibrium, kinetic and structural studies of AAZTA complexes with Ga^3+^, In^3+^ and Cu^2+^. Eur J Inorg Chem. 2013;(1):147–62.

[CR2] Baum RP, Kulkarni HR (2012). Theranostics: from molecular imaging using Ga-68 labeled tracers and PET/CT to personalized radionuclide therapy - the Bad Berka experience. Theranostics..

[CR3] Baum RP, Rösch F (2013). Theranostics, Gallium-68, and other radionuclides.

[CR4] Berry DJ, Ma Y, Ballinger JR (2011). Efficient bifunctional gallium-68 chelators for positron emission tomography: tris (hydroxypyridinone) ligands. Chem Commun (Camb).

[CR5] Boros E, Ferreira CL, Cawthray JF (2010). Acyclic chelate with ideal properties for ^68^Ga PET imaging agent elaboration. J Am Chem Soc.

[CR6] Boros E, Ferreira CL, Yapp DTT (2012). RGD conjugates of the H_2_dedpa scaffold: synthesis, labeling and imaging with ^68^Ga. Nucl Med Biol.

[CR7] Eisenwiener KP, Prata MIM, Buschmann I (2002). NODAGATOC, a new chelator-coupled somatostatin analogue labeled with [^67/68^Ga] and [^111^In] for SPECT, PET, and targeted therapeutic applications of somatostatin receptor (hsst_2_) expressing tumors. Bioconjug Chem.

[CR8] Farkas E, Nagel J, Waldron BP (2017). Equilibrium, kinetic and structural properties of gallium (III) and some divalent metal complexes formed with the new DATA^m^ and DATA^5m^ ligands. Chem - A Eur J.

[CR9] Filosofov BD V, Loktionova NS, Rösch F. A ^44^Ti/^44^Sc radionuclide generator for potential application of ^44^Sc-based PET-radiopharmaceuticals. 2010;156:149–156.

[CR10] Kerdjoudj R, Pniok M, Alliot C (2016). Scandium (III) complexes of monophosphorus acid DOTA analogues: a thermodynamic and radiolabelling study with ^44^Sc from cyclotron and from a ^44^Ti/^44^Sc generator. Dalt Trans.

[CR11] Khawar A, Eppard E, Sinnes JP, et al. [^44^Sc]Sc-PSMA-617 biodistribution and dosimetry in patients with metastatic castration-resistant prostate carcinoma. Clin Nucl Med February. 2018;1. 10.1097/RLU.0000000000002003 29485430

[CR12] Lebedev NA, Novgorodov A, Misiak R, Brockmann J, Rösch F (2000). Radiochemical separation of no-carrier-added Lu-177 as produced via the Yb-176 (n ,γ) Yb-177 → ^177^Lu process. Appl Radiat Isot.

[CR13] Manzoni L, Belvisi L, Arosio D (2012). Synthesis of Gd and ^68^Ga complexes in conjugation with a Conformationally optimized RGD sequence as potential MRI and PET tumor-imaging probes. ChemMedChem..

[CR14] Nagy Gábor, Szikra Dezső, Trencsényi György, Fekete Anikó, Garai Ildikó, Giani Arianna M., Negri Roberto, Masciocchi Norberto, Maiocchi Alessandro, Uggeri Fulvio, Tóth Imre, Aime Silvio, Giovenzana Giovanni B., Baranyai Zsolt (2017). AAZTA: An Ideal Chelating Agent for the Development of 44 Sc PET Imaging Agents. Angewandte Chemie International Edition.

[CR15] Pfister J, Summer D, Rangger C (2015). Influence of a novel, versatile bifunctional chelator on theranostic properties of a minigastrin analogue. EJNMMI Res.

[CR16] Price EW, Orvig C (2014). Matching chelators to radiometals for radiopharmaceuticals. Chem Soc Rev.

[CR17] Pruszyński M, Loktionova NS, Filosofov DV, Rösch F (2010). Post-elution processing of ^44^Ti/^44^Sc generator-derived ^44^Sc for clinical application. Appl Radiat Isot.

[CR18] Roesch F (2012). Scandium-44: benefits of a long-lived PET radionuclide available from the ^44^Ti/^44^Sc generator system. Curr Radiopharm.

[CR19] Rösch F, Herzog H, Qaim SM (2017). The beginning and development of the Theranostic approach in nuclear medicine , as exemplified by the radionuclide pair ^86^Y and ^90^Y. Pharmaceuticals..

[CR20] Schuhmacher J, Klivenyi G, Hull WE (1992). A bifunctional HBED-derivative for labeling of antibodies with ^67^Ga, ^111^In and ^59^Fe. Comparative biodistribution with ^111^In-DPTA and ^131^I-labeled antibodies in mice bearing antibody internalizing and non-internalizing tumors. Nucl Med Biol.

[CR21] Seemann J, Waldron B, Parker D, Roesch F (2017). DATATOC: a novel conjugate for kit-type ^68^Ga labelling of TOC at ambient temperature. EJNMMI Radiopharm Chem.

[CR22] Seemann J, Waldron BP, Roesch F, Parker D (2015). Approaching “kit-type” labelling with ^68^Ga: the DATA chelators. ChemMedChem..

[CR23] Tsionou MI, Knapp CE, Foley CA (2017). Comparison of macrocyclic and acyclic chelators for gallium-68 radiolabelling. RSC Adv.

[CR24] Waldron BP, Parker D, Burchardt C (2013). Structure and stability of hexadentate complexes of ligands based on AAZTA for efficient PET labelling with gallium-68. Chem Commun (Camb)..

[CR25] Zhernosekov KP, Filosofov DV, Baum RP (2007). Processing of generator-produced medical application for. Nucl Med.

